# TopoDB: a novel multifunctional management system for laboratory animal colonies

**DOI:** 10.1093/database/baaa098

**Published:** 2020-11-18

**Authors:** Adam Renschen, Atsuko Matsunaga, Jorge R Oksenberg, Adam Santaniello, Alessandro Didonna

**Affiliations:** Weill Institute for Neurosciences, Department of Neurology, University of California San Francisco, San Francisco, CA 94158, USA; Weill Institute for Neurosciences, Department of Neurology, University of California San Francisco, San Francisco, CA 94158, USA; Weill Institute for Neurosciences, Department of Neurology, University of California San Francisco, San Francisco, CA 94158, USA; Weill Institute for Neurosciences, Department of Neurology, University of California San Francisco, San Francisco, CA 94158, USA; Weill Institute for Neurosciences, Department of Neurology, University of California San Francisco, San Francisco, CA 94158, USA

## Abstract

Animal models are widely employed in basic research to test mechanistic hypotheses in a complex biological environment as well as to evaluate the therapeutic potential of candidate compounds in preclinical settings. Rodents, and in particular mice, represent the most common *in vivo* models for their small size, short lifespan and possibility to manipulate their genome. Over time, a typical laboratory will develop a substantial number of inbred strains and transgenic mouse lines, requiring a substantial effort, in both logistic and economic terms, to maintain an animal colony for research purposes and to safeguard the integrity of results. To meet this need, here we present TopoDB, a robust and extensible web-based platform for the rational management of laboratory animals. TopoDB allows an easy tracking of individual animals within the colony and breeding protocols as well as the convenient storage of both genetic and phenotypic data generated in the different experiments. Altogether, these features facilitate and enhance the design of *in vivo* research, thus reducing the number of necessary animals and the housing costs. In summary, TopoDB represents a novel valuable tool in modern biomedical research.

**Database URL**: https://github.com/UCSF-MS-DCC/TopoDB

## Background

Animal models are broadly employed in modern biomedical research to recapitulate specific aspects of human physiology and preclinical testing of therapeutic reagents (1). Rodents, especially *Mus musculus* and *Rattus norvegicus*, are among the most widely used *in vivo* models for their small size, short lifespan and genetic similarity with humans (2). Recent technological advances in our ability to manipulate the mouse genome at the single nucleotide level resulted in an increasing number of genetically modified lines (including transgenic, knock-out and knock-in animals, either constitutive or conditional), often used simultaneously in single projects (3, 4). The management of each specific line requires a substantial effort in terms of breeding planning, animal husbandry and genotyping. Furthermore, experimental cohorts usually contain double-digit individuals to assure the statistical robustness and reproducibility of the results, resulting in large amounts of genetic and phenotypic data generated within each experiment (5).

Careful organization and storage of animal colony data are critical to avoid errors that invalidate experiments, which inevitably result in the wasteful use of animals and resources. In addition, the possibility to structure data accessibility to multiple users allows a tighter control over the existing animal colonies, leading to improved breeding strategies and fewer animals needed for both colony maintenance and experimental purposes, in line with the principles of the 3Rs for animal research (Replacement, Reduction and Refinement) (6). Existing solutions for managing these data vary widely. Easily adopted tools such as paper notebooks and spreadsheets are error-prone and do not scale well to handle large data or complex colony structures. Electronic databases also exist, either free-ware or commercial. Several open-access tools have been developed over the years (7–10). Although generally affordable, they often reflect the specific needs of the developers, hence displaying limited flexibility in different research settings. Conversely, commercial animal colony software, while generally robust and scalable, is frequently expensive and cumbersome to use, with steep learning curves that discourage their use in standard research environments.

To address the need for a robust, inexpensive, easy-to-use and scalable animal colony data management platform, here we present TopoDB (from ‘topo’, the Italian word for ‘mouse’), a web-based, open-source laboratory animal management system. This tool bridges the gap between currently existing in-house applications and available commercial solutions. TopoDB offers a rational and trackable storage of small to large amounts of inventory, genetic and phenotypic data in tune with the increasing regulatory pressures. Moreover, it allows the option to perform preliminary data analysis and result visualization. As proof of principle, we show the handling of clinical scores from mice after induction of experimental autoimmune encephalomyelitis (EAE), an *in vivo* model of multiple sclerosis (MS) that we extensively use in our research (11–14).

## Methods

### Database overview

TopoDB is a web-based application built using the open-source website framework Ruby on Rails, with MySQL as the database component. It was developed in Mac OS X and Linux (Ubuntu) environments. Additional open-source software libraries were incorporated to enhance design (Bootstrap 4), functionality (Best-In-Place), data integrity (Paper Trail, CanCan) and security (Devise). Technical requirements for deployment are relatively modest, and our production deployment machine uses an AMD Opteron 1.8-GHz CPU and 16-GB RAM.

The initial production deployment of TopoDB was in January 2020, managing over 600 animals across 20 wild-type and transgenic lines in 200 cages spread over 2 separate facilities.

### Database building strategy

**Figure 1.**
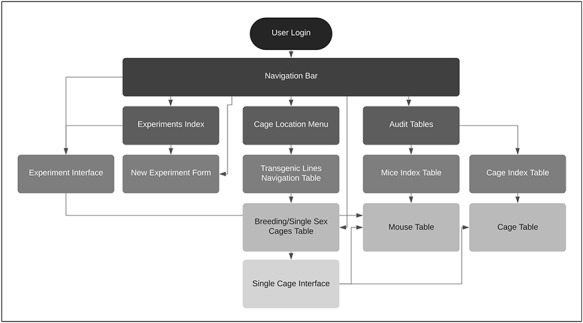
TopoDB website architecture.

User feedback guided the design and development of the TopoDB application (Figure 1). Our approach was to gather first core colony management feature requirements from multiple users with experience in animal research. Primary concerns were accurate recording of mouse-level phenotype and genotype data, and a way to record and audit each mouse’s history, including recording the birth of pups, biopsy collection information and transfer of mice between cages. After translating these requirements into technical solutions, users were presented with design options for all major interface components including data entry methods and web page design. The development process involved prototyping features based on user input, then deploying a new build of the application to a dedicated user acceptance platform for testing. The feedback generated by user testing exposed design flaws and software bugs, which were then remedied by the developer, after which an updated version of the application was deployed for additional testing. This process was repeated until the users were satisfied that each feature was useful and working as intended

### Animals

C57BL/6J female mice of 8 weeks of age were purchased from The Jackson Laboratory (stock number: 000664). After arrival, mice were maintained in a specific-pathogen-free (SPF) facility. The animals were housed (five mice per cage) in a room with 12:12-h light/dark cycle. Standard mouse chow and water were provided *ad libitum*. All animal procedures were performed in compliance with experimental guidelines approved by the University of California San Francisco (UCSF) Committee on Animal Research (CAR).

### EAE induction

Mice were randomly assigned to each experimental group and acclimated for 1 week before use. Active EAE was induced following previously published procedures (13, 15). Briefly, mice were injected subcutaneously with 100 μg of murine myelin oligodendrocyte glycoprotein (MOG) 35-55 (EZBiolab), in complete Freund’s adjuvant (CFA) with 4 mg/mL of *Mycobacterium tuberculosis* (Difco Laboratories). Mice also received 400 ng of pertussis toxin (List Biological Laboratories) intraperitoneally at the time of immunization and 48 h later. Control mice were mock injected with everything except the MOG peptide. For all experiments, animals were observed daily, and clinical signs were assessed as follows: 0, no signs; 1, decreased tail tone; 2, mild monoparesis or paraparesis; 3, severe paraparesis; 4, paraplegia; 5, quadriparesis and 6, moribund or death. All scores were assigned blindly to the treatment of the mice.

## Results

### User interface

**Figure 2.**
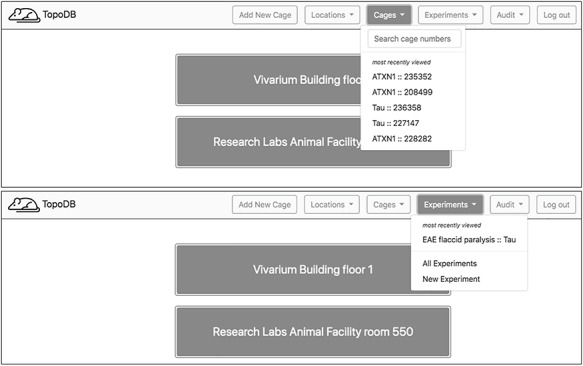
Upon login, a home page showing the top level of cage organization—Locations—is shown. A navigation bar at the top of each page enables users to quickly access recently visited cages and experiments. A search box also assists in finding specific cages.

Using TopoDB should be a familiar experience for users of modern Internet and spreadsheet applications. The user interface is clean and uncluttered, utilizing the principles of modern material web design. A primary navigation bar (‘navbar’) contains dropdown menus for easy navigation between the main areas of concern: locations, cages, experiments and data auditing (Figure 2). In the cages menu, a search bar provides a fast way to find cages by their cage number as well as a list of hyperlinks to the five most recently viewed cages. Other primary functions, such as creating new experiments and viewing database updates, are also accessible through navbar menus.

Navigation from the home page to a single cage’s page can also be done via hyperlinks on each view. On the home page, users may select a location, usually a building or room. The proceeding view displays a table of transgenic lines and summary information about each line’s population. Each table row in turn links to a page displaying more granular information about that line’s population including tables of individual cages grouped by function, either breeding or adult single-sex cages. From this view, individual cages may be selected.

### Locations

Within TopoDB, cages are organized primarily by their location. This property is set on the individual cage level at the time the cage is first created and may be edited later. For maximum flexibility, the location value is free text naming anything from a city, building, room, laboratory or even shelf. When creating a cage, the location field displays a dynamically generated list of unique location values by examining all existing cages. Users may choose from this list or input a new value.

### Cages

**Figure 3.**
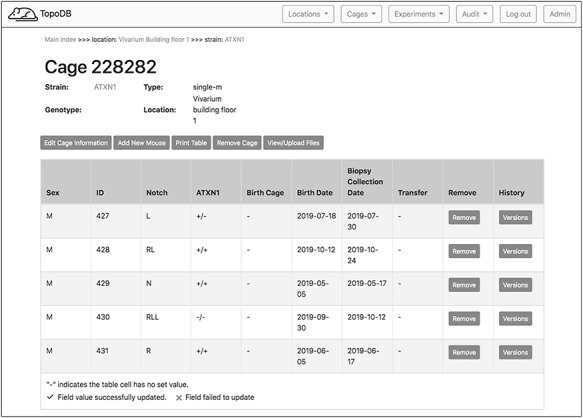
The single cage view in TopoDB summarizes all the principle information about the animals housed in each cage of the colony. This includes the sex, the unique identification number, the corresponding identification code (such as ear punches or ear tags), the date of birth and the biopsy collection date. The possible transfer date from or to another cage is also displayed in this interface.

The main interface for inputting and editing mouse-level data is the single cage view (Figure 3). This page displays cage metadata and a row of buttons that either activate data entry forms or automate other frequently performed tasks. A spreadsheet-like table represents the cage’s mice. For each animal in a certain cage, it is possible to specify its unique alphanumeric identifier, the sex, the strain, the genotype, a biopsy collection date and the date of birth. Data points may be directly input by activating (clicking on) a table cell and either entering a value or choosing one from a select menu of predetermined options. Once a value is input, it is validated and saved. Data validation results are displayed within the table cell, showing users whether their inputs were accepted. These data entry transactions are performed asynchronously by the browser, so users can update any number of data points for all mice without refreshing the page.

The cage view also contains dedicated buttons activating essentials functions in the management of animals. The cage data can be downloaded in a .csv file as well as either printed or saved as a .pdf file by clicking the appropriate button. To record the transfer of a mouse to a different cage, users can access a menu of existing cages by activating the ‘transfer’ cell of the cage table. This list is automatically populated with appropriate target cages that match a mouse’s sex and transgenic line. By selecting a cage from the menu, the mouse is assigned to the new cage. A successful transfer will be noted with a green check mark in the ‘transfer’ table cell.

**Figure 4.**
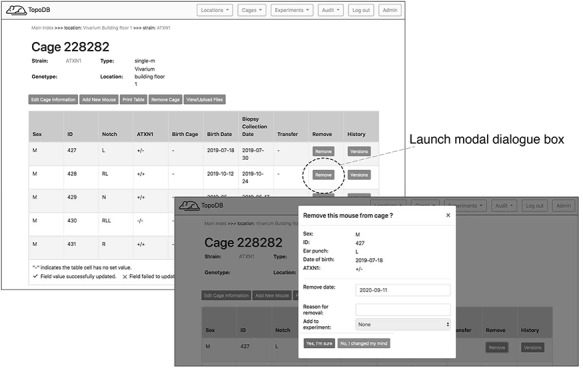
Dedicated buttons in the single cage view activate specific functionality. Here the form for recording the removal of a mouse from the colony (for experimentation or other reasons) is presented as a modal overlaying the single cage view.

**Figure 5.**
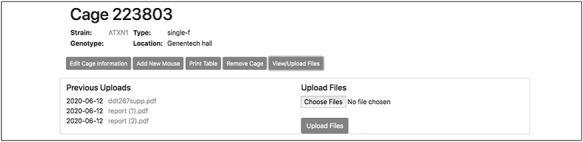
This single cage interface allows attaching valuable metadata that are associated with the animals in the cage. They can include the genotyping results of a specific transgenic line, the vendor’s documentation for founder mice or the cryopreservation details.

To record the permanent removal of a mouse from the colony, clicking on the ‘Remove mouse’ button activates a modal dialog form (Figure 4). Here, a user can input the date and reason for the animal’s removal. This dialog form can be also used to assign an animal to an existing experiment, if one is available, by selecting from the ‘Add to experiment’ select menu. The cage interface also supports uploading external files of any format (such as .pdf, .jpg, .tiff, and .txt) to ‘attach’ to the cage. The dialog form is activated by the button ‘View/Upload Files’. Within this dialog, users may also see a list of prior file uploads and select files for previewing by clicking the filename (Figure 5).

### Experiments

The possibility to store and easily retrieve the data points collected within the different experiments is a central need for all *in vivo* researchers. To this end, we have implemented in TopoDB the ‘Experiments’ interface where the user can create summary pages of the experiments performed over time and univocally link to each of them the animals present in the colony. In addition, the experimental measures collected for every animal can be organized in practical tables, using a system of X-Y coordinates. Lastly, mean values and relative standard errors (SEs) are automatically calculated for each experimental cohort, in order to facilitate a preliminary assessment of the effects of specific treatments or genotypes on the phenotype of interest.

**Figure 6.**
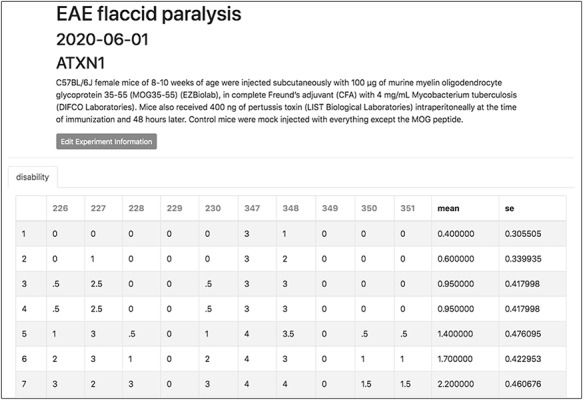
The experiment interface of TopoDB allows the efficient organization and storage of experimental data points. In this example, the EAE scores collected on a cohort of 10 mice up to 11 days postimmunization are organized in a spreadsheet format. The individual mouse ID numbers are displayed on the top row. Mean values and SEs are also automatically calculated for each day.

**Figure 7.**
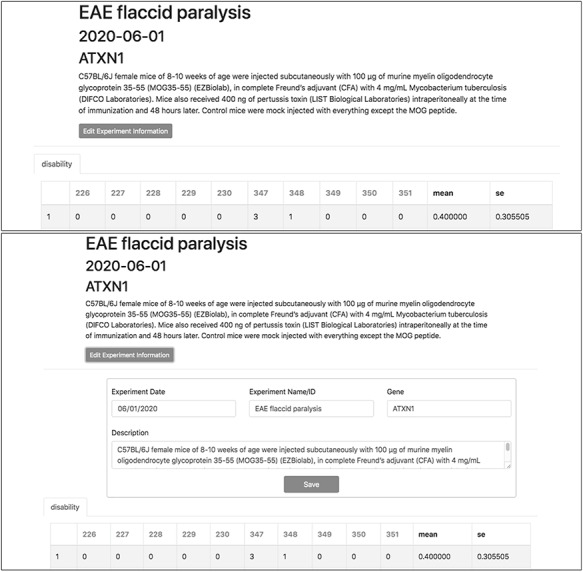
Experiment metadata can be edited by clicking the ‘Edit Experiment Information’ button in the experiment interface. ‘Accordian’-like elements, invisible until activated by the user, keep pages uncluttered, avoid time-consuming navigation and provide utility.

To demonstrate a practical application of this feature, here we show the organization and analysis of clinical scores from mice upon EAE, a disease model recapitulating several aspects of MS pathology. After immunization with encephalitogenic peptides, EAE mice develop over time symptoms of flaccid paralysis that are daily quantified according to established scoring systems. Within each experiment, cohorts of at least 10 mice are followed for 30 days, thus generating a data set of 600 data points between EAE and control animals. TopoDB allows a convenient tabulation of such amount of data (Figure 6). The display of mean EAE scores for each day helps in the analysis of key parameters of this model such as disease onset (Day 8 postimmunization), disease peak (Day 16 postimmunization) and the beginning of the chronic phase (Day 20 postimmunization). Moreover, important details associated with each experiment (such as the specific protocol adopted, the person who conducted the experiment or the reagent lots employed) can be added through the ‘Edit Experiment Information’ button (Figure 7).

### Data auditing

**Figure 8.**
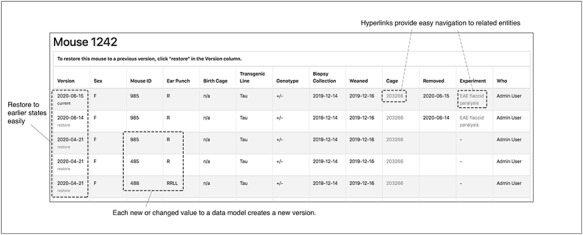
The audit interface records all the modifications submitted for each mouse in TopoDB in chronological order. This feature allows the easy identification of incorrectly input data and the efficient recovery of the correct information from previous versions stored in the database.

To enhance the accuracy of data entry, the TopoDB user interface includes an intuitive means of viewing the history of all data transactions of each mouse and cage. The database component of TopoDB employs the Paper Trail library to log all changes to mice, cages and experiments. When an update to one of these objects is successfully committed to the database, information about the previous state, including which values were updated and the user who authored the update, is recorded in a separate table. On the web interface, a page containing a table that displays a chronologically ordered list of states or ‘versions’ of the mouse is provided under the ‘Audit’ navbar menu (Figure 8). Users can easily undo data entry errors by reverting a record to a previous state by selecting it from the table. This function is particularly useful to amend mistakes in genotype assignment of new litters.

### Website and database administration

Centralized storage of data in a relational database creates a durable, highly organized electronic record of all colony data. Database files are easily copied and backed up, avoiding the single-point-of-failure weakness frequently found in paper-based record keeping. After initial deployment to a server, TopoDB can be operated without specialized website administration and database programming skills. An easy to use web interface is available to admin-level users to perform most common website and database administration tasks (Table 1). Users with sufficient authorization may approve new users, manage existing users’ accounts and even directly update database records.

**Table 1.** Overview of the different administrator and user privileges.

	Approve/remove	Direct		
	users and elevate	database	Insert/update	Audit
Role	permissions	access	data	data
Admin	Yes	Yes	Yes	Yes
User	No	No	Yes	Yes

## Discussion

The initial production deployment of the TopoDB platform was in January 2020 and is the primary tool for managing over 600 animals across 20 wild-type and transgenic lines in 200 cages spread over 2 separate facilities. The core design was created to meet the needs of our laboratory, but the platform can be adapted for use by any facility needing a flexible, low-cost solution for managing animal data. The TopoDB platform was created using open-source software and is released free of charge.

The Technology Acceptance Model (16) suggests that perceived ease of use and perceived usefulness are critical factors in users’ adoption of a technology. In contrast with other laboratory information management system (LIMS) programs, TopoDB has an uncluttered user interface design that is simple and intuitive. Navigation logically progresses from general (entire colony) to specific (individual cage). On each page, appropriate summary information about the population is displayed in tables and/or interactive graphics, while a ‘breadcrumb’ menu shows the current page’s position in the directory structure and provides additional site navigation. Users familiar with the Internet and/or spreadsheet programs such as Microsoft Excel or Apache OpenOffice Calc should face a minimal learning curve, accelerating onboarding of laboratory staff.

Other colony management programs frequently have limited customization options or require costly subscriptions to use desirable features (Table 2). TopoDB is built entirely with open-source software and can be extended and adapted to suit the needs of other laboratories, and even to manage other types of laboratory animals (either vertebrate or invertebrate), provided that they are individually identified.

**Table 2.** Feature comparison with other colony management software.

Software	Mouse phenotyping	Cage transfers	Experimental data storage	File upload	Audit logs	Admin interface
TopoDB	Yes	Yes	Yes	Yes	Yes	Yes
Softmouse	Yes	Yes	Paid	Paid	Paid	Paid
MausDB	Yes	Yes	No	Yes	No	No
RodentSQL	Yes	No	No	No	No	Yes
mLIMS	Paid	Paid	No	Paid	Paid	Paid

Development to enhance TopoDB continues. To ease DevOps concerns around web site deployment and to increase avenues of distribution, we will create a containerized version to run in the Docker ecosystem and be distributed via the DockerHub repository. Additionally, we will update the codebase to independently track multiple animal colonies within a single deployment of the platform and to scope user access and authorization to these colonies independently. Other planned features include report generation, both of veterinary information and financial costs associated with animal care and housing. Finally, we plan to strengthen the data analysis features by extending the number of statistical tests that can be performed in TopoDB and by implementing data visualization tools. In summary, TopoDB represents a valuable option for any laboratory in the academia or industry conducting *in vivo* research at any scale in terms of colony size and transgenic line numbers.

## onflict of interest

None declared.
